# Potential of Polymeric Films Loaded with Gold Nanorods for Local Hyperthermia Applications

**DOI:** 10.3390/nano10030582

**Published:** 2020-03-23

**Authors:** Álvaro Cárcamo-Martínez, Juan Domínguez-Robles, Brónach Mallon, Md. Taifur Raman, Ana Sara Cordeiro, Steven E. J. Bell, Eneko Larrañeta, Ryan F. Donnelly

**Affiliations:** 1 School of Pharmacy, Queen’s University Belfast, 97 Lisburn Road, Belfast BT9 7BL, UKj.dominguezrobles@qub.ac.uk (J.D.-R.); bmallon14@qub.ac.uk (B.M.); a.cordeiro@qub.ac.uk (A.S.C.); e.larraneta@qub.ac.uk (E.L.); 2 School of Chemistry and Chemical Engineering, Queen’s University Belfast, Belfast BT9 5AG, UK; t.rahman@qub.ac.uk (M.T.R.); s.bell@qub.ac.uk (S.E.J.B.)

**Keywords:** non-melanoma skin cancer, hyperthermia, gold nanorods, polymeric films, near infrared light, heating studies

## Abstract

Current strategies for the treatment of superficial non-melanoma skin cancer (NMSC) lesions include topical imoquimod, 5-fluorouracil, and photodynamic therapy. Although these treatments are effective, burning pain, blistering, and dermatitis have been reported as frequent side effects, making these therapies far from ideal. Plasmonic materials have been investigated for the induction of hyperthermia and use in cancer treatment. In this sense, the effectiveness of intratumorally and systemically injected gold nanorods (GnRs) in inducing cancer cell death upon near-infrared light irradiation has been confirmed. However, the in vivo long-term toxicity of these particles has not yet been fully documented. In the present manuscript, GnRs were included in a crosslinked polymeric film, evaluating their mechanical, swelling, and adhesion properties; moreover, their ability to heat up neonatal porcine skin (such as a skin model) upon irradiation was tested. Inclusion of GnRs into the films did not affect mechanical or swelling properties. GnRs were not released after film swelling, as they remained entrapped in the polymeric network; moreover, films did not adhere to porcine skin, altogether showing the enhanced biocompatibility of the material. GnR-loaded films were able to heat up the skin model over 40 °C, confirming the potential of this system for non-invasive local hyperthermia applications.

## 1. Introduction

Incidence of non-melanoma skin cancers (NMSC) is increasing worldwide. Reports show that in the UK, 47,000 new NMSC cases are diagnosed every year, and since the early 90s, incidence rates have increased by more than two-and-a-half times (163%) [1]. A similar trend has been reported in Germany [2], Denmark [3], Canada [4], the USA [5], and Australia [6], among other countries. Although death is rare, treatment of NMSC results in a considerable burden on healthcare systems [7,8,9]. For superficial lesions, non-invasive approaches involving topical imoquimod, 5-fluorouracil, and photodynamic therapy are preferred [10,11,12]. Although effective for this type of lesion, side effects such as itching, burning pain, erythema, dermatitis, blistering, necrosis, and pruritus have been reported as highly frequent [10,13], making these treatments unpleasant and far from ideal.

The primary and adjunctive treatment of cancers by induced hyperthermia is a well-established but burgeoning field of medical research. In this sense, temperatures in tumor-loaded tissues are elevated to more than 43 °C by selective or non-selective application of microwave, radio, ultrasound, alternating magnetic, infrared, or visible radiation [14,15], triggering either necrosis or apoptosis [16,17]. In the first case, when the temperature is raised above 50 °C, it disrupts the plasma membrane, causing leaking of cellular components, and thereby inflammation, metastasis, and harm to surrounding normal tissue could take place [16]. On the other hand, induction of apoptosis takes place when the temperature increases to 43–50 °C. Apoptosis is a standard part of the cell cycle, although it is inhibited in cancer cells to allow for unregulated growth. Hence, as it is an internal process, no extracellular leakage or inflammation is triggered [17], and therefore, hyperthermia should be modulated towards an apoptotic response.

Interest in gold nanorods (GnRs) as thermal agents is due to their ability to emit heat by absorbing near infrared light, on the basis of the phenomenon of localized surface plasmon resonance (LSPR) [18,19]. The GnR spectra exhibit two plasmon resonances: the transverse plasmon is the result of excitation across the nanorod diameter and the longitudinal plasmon is due to excitation along the nanorod length [20]. Hence, the diameter of GnRs can be an important differentiation factor for their applications; scattering becomes dominant for thicker GnRs, and so they are favorable for biolabeling and contrast purposes. On the other hand, absorption predominates in thin GnRs, making them well-suited for photothermal applications that require high photon-to-heat conversion efficiency [21,22].

Investigations have shown the effectiveness of photothermal therapy using intratumorally and systemically injected GnRs in inducing cancer cell death [16,18,23,24]. However, research addressing the safety and toxicity of GnRs is still controversial [23,25,26,27]. The surface chemistry of GnRs has been recently linked to toxicity in cultured cells and mice [23]. This could be attributed mainly to the surfactant used in the synthesis of GnRs, cetyltrimethylammonium bromide (CTAB), which has been associated with cytotoxicity at micromolar concentrations [18,28]. Hence, strategies to increase biocompatibility have been followed, such as functionalization, of the surfaces of GnRs with polymers, including poly(acrylic acid), poly(allylamine) hydrochloride, and poly(styrene)sultanate, or by exchanging CTAB with a less toxic ligand, such as mercaptohexadenoic acid or poly(ethylene)glycol [16,21,23,28]. Furthermore, surface modification of GnRs with manganese dioxide or anti-nucleolin aptamer (AS1411), among others, has been explored to increase the performance of photothermal conversion [29]. However, the in vivo long-term toxicity of these functionalized GnRs has not yet been fully documented.

Until further and high-level evidence regarding the safety and toxicological profile of GnRs is gathered, approaches where body exposure to these particles is limited or avoided should be used. In the present work, CTAB-GnRS were prepared by the seed-mediated method [22] and functionalized with thiolated poly(ethylene) glycol (mPEG-SH) [30]. Both types of GnRs were characterized and then included in polymeric crosslinked films, evaluating their effect on film structural properties through mechanical, swelling, and thermal analyses. Finally, the heating capacity of films upon laser irradiation and the transfer of heat to a skin model were tested to evaluate the possible application of GnR-loaded films for local skin hyperthermia.

## 2. Materials and Methods

### 2.1. Materials

Gantrez^®^ S-97, copolymer of methyl vinyl ether and maleic acid (PVME/MA), with a molar mass of 1,200,000, was donated by Ashland (Barcelona, Spain). Tetrachloroauric acid (HAuCl_4_)_,_ cetyltrimethylammonium bromide (CTAB), sodium borohydride (NaBH_4_), L-ascorbic acid, poly(ethylene glycol) (PEG) 200 Da, and poly(ethylene glycol)methyl ether thiol (mPEG-SH) were purchased from Sigma-Aldrich (St. Louis, MO, USA). The 5-Bromosalicylic acid (5-BrSA) and silver nitrate (AgNO_3_) were purchased from Alfa Aesar (Lancashire, UK). Neonate porcine skin from stillborn piglets was used for heating studies, with full-thickness skin samples being obtained in less than 24 h post-mortem, rinsed in phosphate buffered saline (PBS), trimmed to a 350 μm thickness using an electric dermatome (Integra Life Sciences^TM^ Padgett Instruments, Princeton, NJ, USA), and cut into 2 × 2 cm^2^ pieces.

### 2.2. Synthesis and Characterization of Gold Nanorods (GnRs)

GnRs were prepared according to a previously reported method [22]. All the glassware was cleaned with aqua regia, rinsed with water, and dried before use. A seed solution was prepared by diluting 2.5 mL of HAuCl_4_ 1 mM with 2.5 mL of purified water and adding 5 mL of CTAB 0.2 M under magnetic stirring. A dilution of 0.6 mL of NaBH_4_ 0.01 M with purified water, making up 1 mL, was injected into the previous mixture under intense stirring (1200 rpm) for 2 min. The final seed solution was kept undisturbed at room temperature for 30 min before use. The growth solution was prepared by mixing 9 g of CTAB and 1.1 g of 5-BrSA with 250 mL purified water at 50–70 °C. After complete dissolution, the mixture was cooled down to 30 °C. Then, 12 mL of AgNO_3_ 4 mM was added and the solution was kept undisturbed at 30 °C for 15 min. Then, 250 mL of HAuCl_4_ 1 mM was added and the solution was stirred for 15 min at 400 rpm. Afterwards, 2 mL of L-ascorbic acid 0.064 M was injected, and the solution was mixed vigorously for 30 s until it became colorless. To prepare the GnRs, 0.8 mL of the seed solution was injected into the growth solution and stirred for 30 s. The mixture, CTAB-GnRs, was left undisturbed at 30 °C for 12 h.

#### 2.2.1. Functionalization of GnRs by Thiolated Poly(ethylene) Glycol (mPEG-SH) and Cetyltrimethylammonium Bromide (CTAB) Removal

Then, 50 mL of CTAB-GnRs was centrifuged at 8500 rpm for 45 min, then the precipitate was resuspended in purified water and centrifuged again in order to remove part of the non-bounded CTAB. The final precipitate was resuspended in 1.2 mL of purified water, mixed with 3.8 mL of mPEG-SH 2 mM, and kept undisturbed at room temperature for 24 h. In order to further wash and completely remove the CTAB, GnRs were dialyzed against water using a regenerated cellulose membrane, namely SnakeSkin™ Dialysis Tubing 7K molecular weight cut-off (MWCO) (ThermoFisher Scientific, Waltham, MA, USA), for 24 h with several replacements of water. After dialysis, samples of pegylated GnRs (PEG-GnRs) were mixed with water to make up a volume of 50 mL.

#### 2.2.2. UV Spectrum of GnRs

CTAB and PEG-GnRs were assessed with a UV microplate FluoStar Optima fluorescence spectrophotometer (BMG Labtech, Ortenberg, Germany) in order to observe their near-infrared (NIR) absorbance properties. All samples were diluted with distilled water in a 1:1 proportion and the UV spectrum between 220 and 1000 nm was recorded.

#### 2.2.3. Transmission Electron Microscopy Images of GnRs

A transmission electron microscope (TEM) was used to measure the length and width of CTAB-GnRs and PEG-GnRs. For this purpose, 10 µL of washed samples was deposited on copper grids and evaluated using a TEM JEM-1400Plus (JEOL, Tokyo, Japan).

#### 2.2.4. Z Potential of GnRs

CTAB and PEG-GnRs suspensions were assessed with a NanoBrook Omni particle sizer and zeta potential analyzer (Brookhaven, New York, NY, USA) to determine their zeta potential, measuring each sample for three cycles.

#### 2.2.5. Raman Spectroscopy Characterization of CTAB and Pegylated GnRs (PEG-GnRs)

GnRs were evaluated with a RamanMicro™ 300 microscope equipped with a RamanStation R3 (PerkinElmer, Waltham, MA, USA) to confirm the replacement of CTAB by mPEG-SH. First, 100 μL of each sample was placed on a glass microscope slide, which was previously covered with aluminium foil, and left to dry. Each sample was exposed 4 times for 5 s each, and the spectrum from 0 cm^−1^ to 3681 cm^−1^ was recorded.

#### 2.2.6. Gold Quantification in GnRs

The gold content in GnR samples was assessed by inductively coupled plasma optical emission spectroscopy (ICP-OES) using a 5100 Synchronous Vertical Dual View ICP (Agilent, Santa Clara, CA, USA). CTAB-GnRs samples measuring 50 mL were centrifuged at 8500 rpm for 45 min, the precipitate was resuspended in purified water, and then centrifuged again to remove part of the non-bounded CTAB and non-reacted materials. In contrast, PEG-GnRs were used directly. Samples measuring 2.5 mL of GnRs were mixed with nitric acid 65%, hydrochloric acid 37%, and purified water, giving a final volume of 50 mL. The solution was kept at 60 °C for 2 h to digest and dissolve all materials. Instruments were calibrated using gold standard solutions and samples were measured afterwards in triplicate.

### 2.3. Fabrication and Characterization of Polymeric Films Loaded with GnRs

Polymeric films containing CTAB-GnRs or PEG-GnRs were fabricated using a mixture (%w/w) of 25% Gantrez^®^ S-97, 10% PEG 200 Da, and q.s. of the concentrated GnRs suspension. For CTAB-GnRs, 50 mL of CTAB-GnRs suspension was centrifuged at 8500 rpm for 45 min and resuspended in the same volume of purified water, centrifuged again, and then concentrated by completing the volume until it reached 5 mL. For PEG-GnRs, the suspension obtained in Section 2 was used directly after dialysis, making up the final volume to 5 mL, as well for comparison purposes. Films without GnRs were also fabricated as controls, using only distilled water instead of GnR suspension. Obtained gels were centrifuged at 5000 rpm for 15 m to remove air bubbles and then cast on a 9 cm^2^ siliconized release liner. Films were left to dry at room temperature for 2 days, cut in approximately 1 cm^2^ pieces and then crosslinked in an oven at 80°C overnight. Films were visualized using digital and a Tabletop TM3030 transmission scanning electron microscope (Hitachi, Tokyo, Japan) and then measured in terms of thickness, area and weight.

#### 2.3.1. Mechanical Properties of Polymeric Films

**Figure 1:**
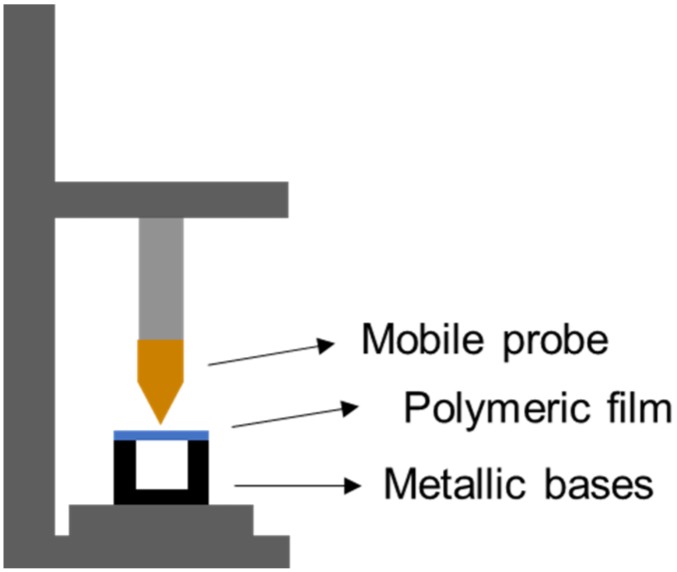
Schematic representation of the texture analyzer setup used to study the mechanical properties of films by recording the force at the breaks of films.

The flexibility of polymeric films and the effect of GnRs on this factor was assessed using a Texture Analyzer TA.XT-Plus (Stable Micro Systems, Surrey, UK), as shown in Figure 1. Briefly, films were placed on top of two aluminium blocks, leaving a distance between the blocks of 7 mm. An aluminium probe was placed on the mobile part of the texture analyzer and moved towards the film at a speed of 1 mm/s while the force and distance travelled by the probe were recorded [31]. The force at the break of each film was determined by looking at the maximum force on the force (N) vs. distance (mm) graph.

#### 2.3.2. Adhesion of Polymeric Films to Neonate Porcine Skin

The adhesive properties of crosslinked films were evaluated using a texture analyzer set in adhesive test mode. Two pieces of dermatomed neonate porcine skin (350 μm thickness each) were placed facing each other’s dermis, pinned into a poly(urethane) foam, and located on the base of the equipment. Films (1 cm^2^) were attached to the mobile probe using double-sided adhesive tape. Before starting the studies, 20 μL of phosphate buffered saline (PBS) were placed on top of porcine skin and the mobile probe was immediately lowered. A force of 5 N for 2 min was applied and then the probe was move upwards at a speed of 0.5 mm·s^−1^. Adhesion was recorded as the maximal force required to detach the sample from the surface of the skin.

#### 2.3.3. Swelling of Polymeric Films on Phosphate Buffered Saline (PBS)

The initial dry weight of the polymeric films was recorded (*m_0_*) before the films were immersed in 5 mL of PBS for 5, 15, 30, and 60 min. At each time point, films were removed, the excess of PBS on the surface was dried using a paper towel, and the films were weighed again (*m_t_*). The swelling index (*SI*) of each film was calculated according to the Equation (1). Digital pictures of films at each time were also taken.

*SI* = (*m_t_* − *m_0_*) × 100/*m* (1)

#### 2.3.4. Thermal Analysis of Polymeric Films

Raw materials used for the fabrication of films (Gantrez^®^ S-97 and PEG 200 Da) and prepared films were evaluated using a 2920 differential scanning calorimeter (DSC) (TA Instruments, Surry, UK). Samples weighing between 3 and 10 mg were placed in aluminium pans and then sealed with an aluminium lid. The samples were heated from 50 to 350 °C, at a rate of 10 °C/min. The heat flow (mW) on samples as a function of temperature was obtained. Same materials and films were also tested using a TGA Q500 thermogravimetric analyzer (TA Instruments, Elstree, UK) by placing 10 mg of sample on aluminium pans. Heating was performed from 50 to 400 °C, increasing temperature at a rate of 10 °C/min. The weight loss (as percentage) of the samples as a function of temperature was obtained.

#### 2.3.5. Fourier Transform Infrared Spectroscopy of Polymeric Films

The spectra of polymeric films were recorded using a Spectrum Two instrument (PerkinElmer, Waltham, MA, USA) equipped with an attenuated total reflectance (ATR) accessory. The spectra were recorded as the mean of 20 scans from 450 to 4000 cm^–1^ using a resolution of 4 cm^–1^, and the % transmittance versus wavelength was recorded. Subsequently, the films were immersed in 10 mL of NaOH 5 M, dried, and the spectrum was again recorded.

#### 2.3.6. GnRs Release from Polymeric Films by Immersion on PBS

Films loaded with GnRs were immersed in 5 mL of PBS for different times (5, 15, 30, and 60 min). At each timepoint, films were removed and the absorbance of an aliquot of PBS (without dilutions) was directly measured on the spectrophotometer. For comparison purposes, a film without GnRs was also tested.

### 2.4. Thermal Studies of GnR-Loaded Polymeric Films

**Figure 2:**
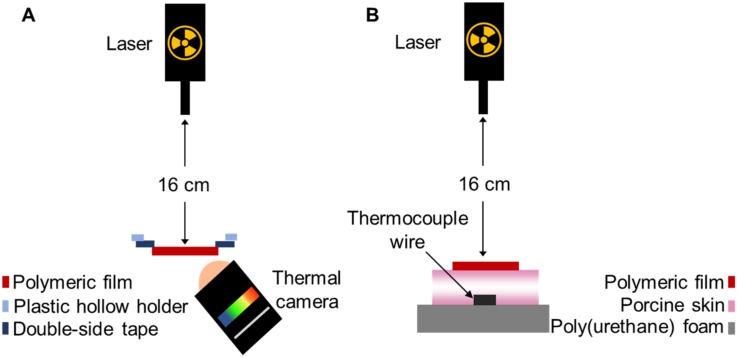
Experimental setup for thermal studies of gold-nanorod-loaded (GnR-loaded) films (**A**). Distance between laser and films was kept at 16 cm. Laser wavelength was 808 nm, with a power of 2W, and film irradiation lasted for 15 s. Experimental setup for thermal studies using neonate porcine skin (**B**). The distance between the laser and polymeric film was kept at 16 cm. Thermocouple wire was placed underneath the porcine skin. The laser wavelength was 808 nm, with a power of 2 W, and films irradiation lasted for 15 s.

The thermal capacity of polymeric films loaded with GnRs was evaluated using a 2W/cm^2^ 808 nm infrared laser (Sunshine-Electronics, Guangdong, China) and a FLIR E8 infrared thermal imaging camera (FLIR, Täby, Sweden), as shown in Figure 2A. Films were placed at a distance of 16 cm from the laser using a sample holder that left 1 cm^2^ of each sample exposed to the laser. The thermal camera was placed at a distance of 2 cm from films. The initial temperature of each film was recorded and then films were irradiated for 3 cycles of 10 s, cooled down between each cycle, and the temperature reached at each time was recorded. A control sample (film without GnRs) was irradiated for 10, 20, and 30 s, with temperatures recorded post-irradiation.

### 2.5. Heating Capacity of Polymeric Films on Neonate Porcine Skin

The capacity of polymeric films loaded with GnRs to heat up neonate porcine skin was evaluated using the setup described in Figure 2B. Two pieces of trimmed skin (350 μm in thickness each) facing each other’s dermis were placed on top of a polystyrene foam and pinned. A thermocouple wire was placed underneath the porcine skin and a thermal camera was used to visualize heating from above as well. A NIR laser (2 W and 808 nm wavelength) was placed above the porcine skin and kept at 16 cm distance. Porcine skin was kept moist by adding 1 mL of fresh PBS and then placing the polymeric films on top. The initial temperature was recorded with both the thermal camera and an OMEGAETTE^®^ HH314A temperature meter (Omega, Manchester, UK). Then, films were irradiated for 15 s and temperatures were reached at 15, 30, 40, 50, 60, and 120 s, which were recorded with both instruments.

### 2.6. Statistical Analyses

Data is shown as mean ± standard deviation (SD) from triplicate measurements, unless otherwise stated. Differences between study groups were assessed for significance using one-way analysis of variance (ANOVA), followed by a multiple comparisons test (Tukey’s test). The threshold for significance was *p* < 0.05. Statistical analysis was performed using Prism 7 (GraphPad Software, San Diego, CA, USA).

## 3. Results and Discussion

### 3.1. Synthesis and Characterization of GnRs

**Figure 3:**
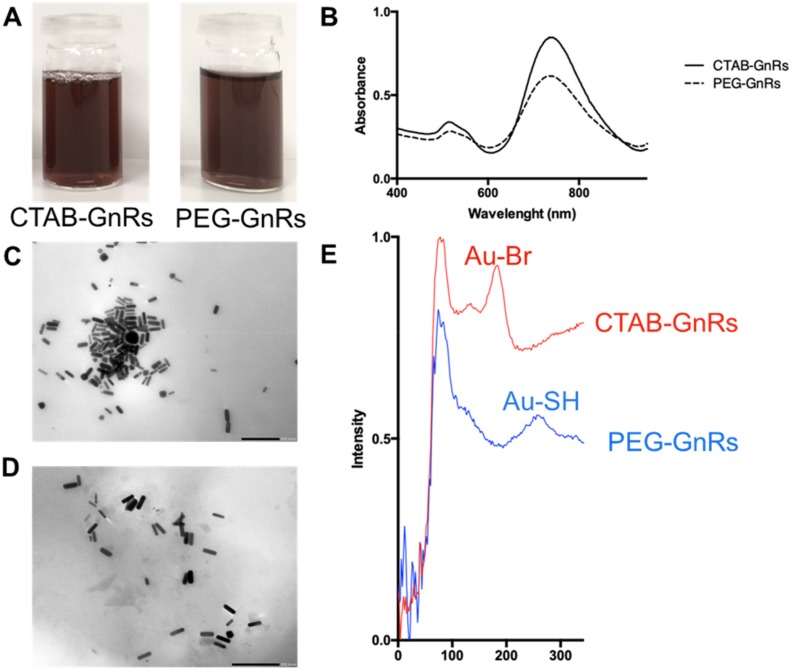
Representative images of GnR samples (**A**). Absorbance spectrum of GnRs within the near-infrared (NIR) spectrum (**B**). Representative transmission electron microscopy (TEM) images of cetyltrimethylammonium bromide GnRs (CTAB-GnRs) (**C**) and pegylated GnRs (PEG-GnRs) (**D**) (scale bar represents 200 nm). Raman spectroscopy of GnRs (**E**).

GnRs were prepared using a previously reported seed-mediated growth method [22], where small gold nanoparticle seeds were added to an aqueous growth solution consisting of a mixture of ionic silver, ionic gold (AuCl_4_^−^), weak reductant (ascorbic acid), and surfactant (CTAB). CTAB has been proven to cause cytotoxicity, complicating the use of GnRs for biomedical applications [23]. Hence, samples of CTAB-GnRs were taken and the CTAB was removed by functionalization with mPEG-SH. Figure 3 shows prepared GnRs, as well as their absorption spectra.

The maximum absorbance in the NIR region for both CTAB and PEG-GnRs was at 735 nm. It can also be seen in the absorbance spectrum that PEG-GnRs had a lower absorbance in both peaks in comparison to the original batch, but no shift in the peaks could be seen. Since the surface plasmon resonance of GnRs depends on their size and aspect ratio [32,33], spectrum results suggest that functionalization with mPEG-SH did not lead to aggregation of particles, and the decrease in absorbance could be attributed to the dialysis process, where some of the particles could have diffused into the dialysis media.

TEM images of the different samples showed that rod-like particles were achieved and that this shape was not changed after PEG functionalization (Figure 3). The dimensions of GnRs are shown in Table 1. CTAB and PEG-GnRs have similar lengths and widths, ranging from 49 to 53 nm in length and 14 to 19 nm in width, making them ideal for hyperthermia purposes, as absorption instead of scattering of NIR light is favored in GnRs below 20 nm width [22,34].

CTAB-GnRs exhibited a positive charge of 40.04 ± 3.98 mV (mean ± S.D., n = 9), due to the cationic nature of the quaternary ammonium bromide moiety of the surfactant [23,30]. The Z-potential of PEG-GnRs decreased to −12.99 ± 3.03 mV, suggesting that CTAB was removed from the particles and replaced by mPEG-SH. Moreover, the results obtained in the Raman spectroscopy showed peaks at 184 and 254 cm^-1^, which have been associated with Au-Br and Au-S bond formation, respectively, also confirming the replacement of CTAB by mPEG-SH [20,30].

The gold content in samples of CTAB and PEG-GnRs was 76.3 ± 4.4 and 41.7 ± 7.3 µg/mL, respectively. Since the theoretical concentration of gold in the pure suspension was 1 mg/mL, the percentage of gold converted into GnRs was 7.63% and 4.17% for CTAB and PEG, respectively, which is within ranges previously reported when the seed-mediated method is used [35,36]. The decrease of metallic gold content in the pegylated samples could be a consequence of the CTAB washing and dialysis step, as some of the GnRs could have been lost during the process.

### 3.2. Preparation and Characterization of Polymeric Films Loaded with GnRs

**Figure 4:**
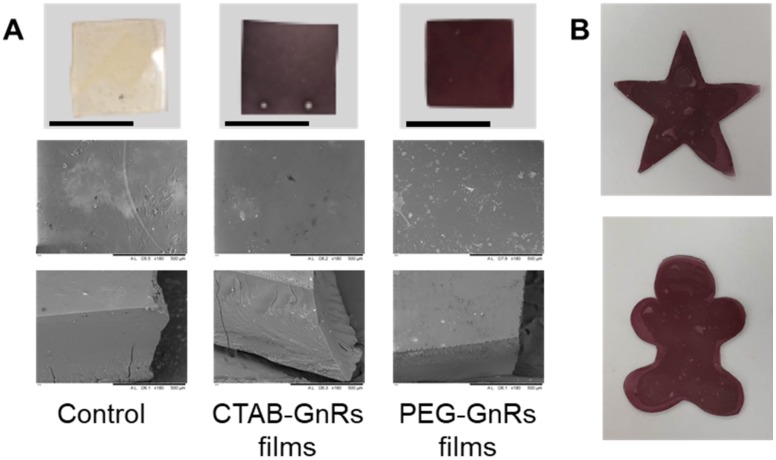
Representative digital and SEM images of polymeric films with GnRs (**A**). Polymeric films cut before crosslinking (**B**) (scale bar on digital images represent 1 cm).

Polymeric films loaded with GnRs were fabricated by casting a mix of Gantrez^®^ S-97, PEG 200 Da, and GnRs onto siliconized paper. After 2 days of drying, films were flexible enough to be removed from the release liner without any damage and were subsequently crosslinked in an oven at 80 °C overnight. In fact, more complex geometries could be prepared by casting the gels in different molds or by simply cutting them before crosslinking, as shown in Figure 4B. Since the morphology of NMSC lesions is variable and does not always exhibit a flat surface [11], films could be tailored to each patient. As happened with the different GnRs samples, differences in color can also be seen on the films due to the different optical properties. On SEM images, it can be seen that polymer and crosslinking agents are fully mixed in a non-porous structure in all films prepared. The thickness, area, and weight of films were recorded (Table 2), and since the same amount of gel was used to prepare the films, similar values were obtained for the aforementioned parameters.

**Figure 5:**
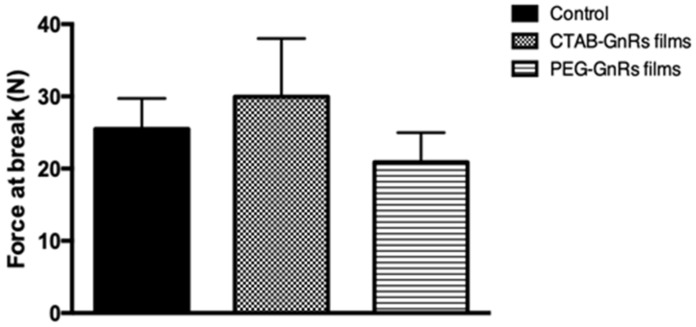
Force at the break of polymeric films (mean ± SD, n = 6).

To investigate whether or not GnRs could have an effect on the mechanical properties of polymeric films, a texture analyzer was used to assess force at the break. Results for this test are shown in Figure 5. When a force of around 30 N was exerted on control films, they broke. Similarly, films including either CTAB or PEG-GnRs showed no significant difference (*p* < 0.05) in this value, suggesting that crosslinking of acid groups of poly(methyl vinyl ether-maleic acid) with alcohol groups of poly(ethylene glycol) was not altered by the presence of GnRs.

**Figure 6:**
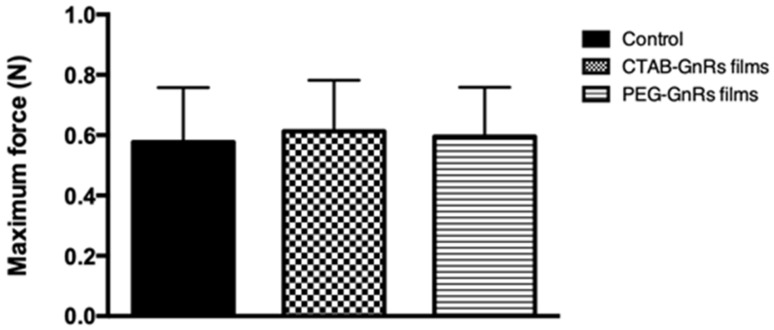
Adhesion studies of polymeric films in neonate porcine skin, where the maximum detachment force was recorded (mean ± SD, n = 3).

When films were placed in contact with porcine skin, it was seen that no adhesion took place, since only 0.60 N was required in all films (control, CTAB, or PEG-GnRs films) for them to be detached, even after 2 min of being in contact with an applied force of 5 N (Figure 6). Significant differences in the detachment force between control and GnR-loaded films were not observed (*p* > 0.05).

**Figure 7:**
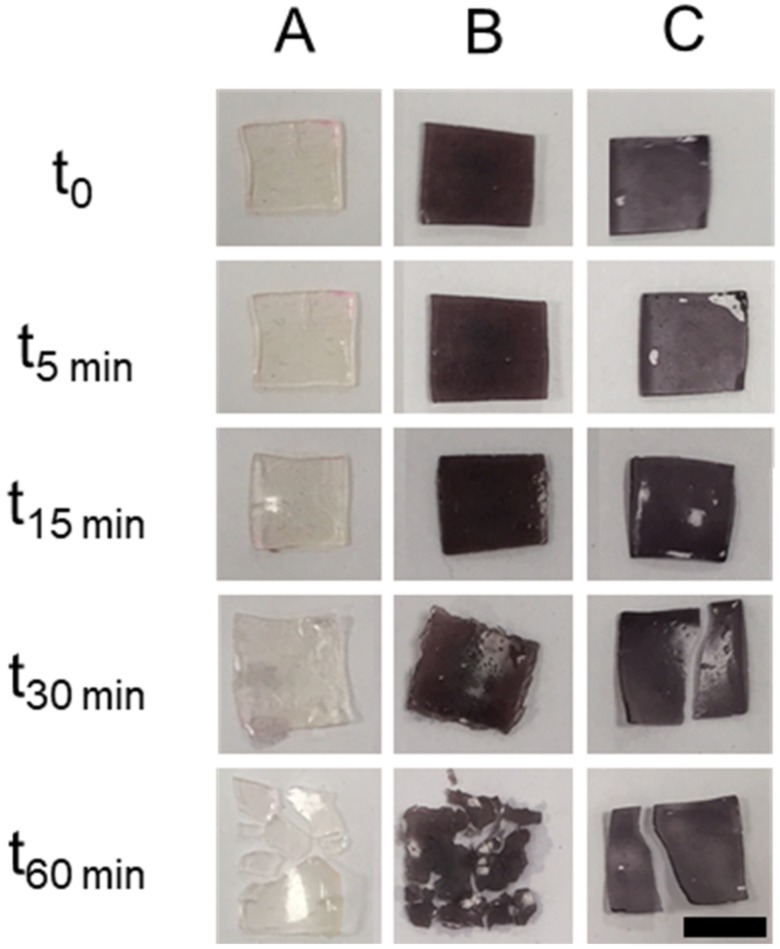
Representative images of films immersed in phosphate buffered saline (PBS) at different times. Control (**A**), CTAB-GnRs film (**B**), and PEG-GnRs film (**C**) (scale bar represents 1 cm).

Films broke into smaller pieces during the swelling studies (Figure 7), and so the percentage of swelling could not be studied. However, pictures at different times show that after 5 and 15 min of immersion, films did not increase in size significantly. After 15 min, films start breaking into smaller pieces. A low molecular weight crosslinking agent was used, and so a highly crosslinked networked was obtained, however the films were quite thin. Then, when exposed to an excess of water, the structure collapsed and broke. The study presented here aimed to observe the swelling behaviour of films only, and so an excess of water was used; however, the setup does not represent what would happen when the films are placed on top of skin.

**Figure 8:**
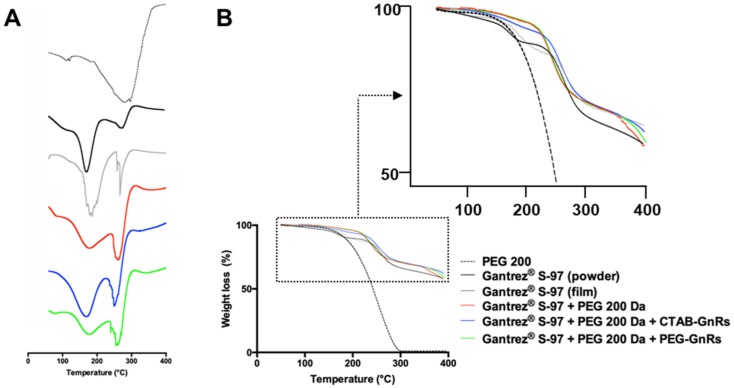
Thermal analysis of PEG 200 Da, Gantrez^®^ S-97, and crosslinked films made with CTAB and PEG-GnRs: Differential scanning calorimetry (DSC) (**A**), thermogravimetric analysis (TGA) (**B**).

DSC curves, showed in Figure 8, obtained for Gantrez^®^ S-97 exhibited a broad peak at 157 °C, which was related to the formation of anhydrides between two acid groups [37,38]. A slight shift to higher temperatures of the Gantrez^®^ S-97 peak can be seen in all films prepared, even in the one prepared only with Gantrez^®^ S-97. This can be attributed to the reaction between alcohol and acid groups, requiring a higher amount of energy to carry on with the reaction [37]. In the case of TGA, it can be seen that PEG 200 Da exhibits a thermal decomposition starting around 160 °C and finishing around 300 °C, which is similar to values previously reported [39]. For the case of Gantrez^®^ S-97, a significant drop in weight loss can be seen around 175 °C, which was associated with the dehydration of the diacid groups. The second drop, starting around 260 °C, was related to decarboxylation and other degradation reactions [38]. Even though both drops in weight loss can be seen in the different prepared films, it is noticeable that Gantrez^®^ S-97, PEG 200, and GnR films were more thermally stable than Gantrez^®^ S-97 pure films, quite likely due to the crosslinking process, since the Gantrez^®^ S-97 onset temperature at 175 °C shifted on all those films to around 230 °C.

**Figure 9:**
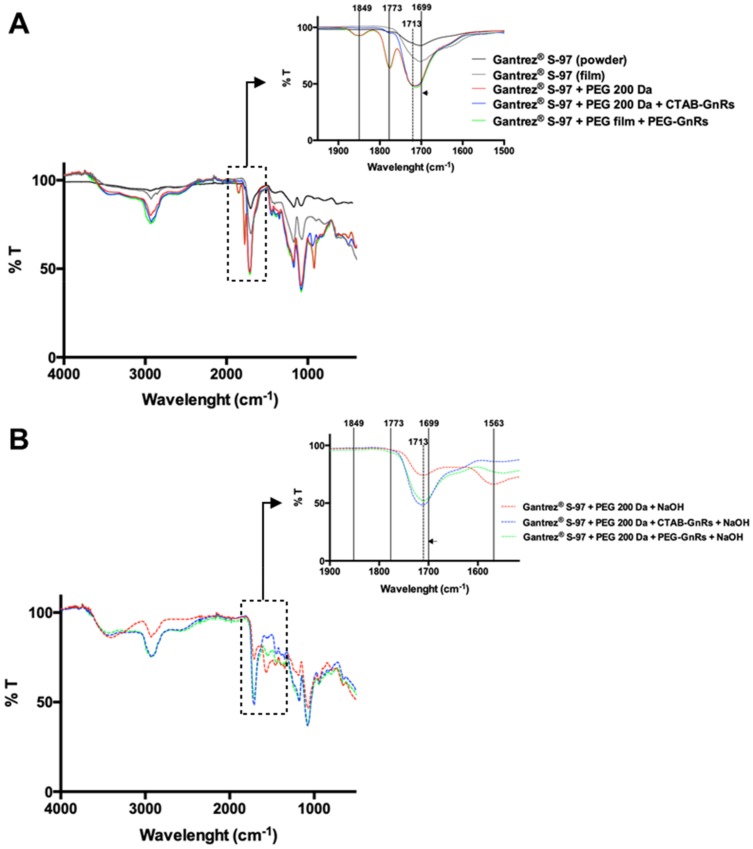
Fourier Transform Infrared Spectroscopy (FT-IR) studies on Gantrez^®^ S-97 (powder) and films before (**A**) and after immersion into NaOH (**B**).

Figure 9A shows the spectra of Gantrez^®^ S-97 powder and films formulated with this excipient, PEG 200 Da, and GnRs. When the carbonyl region of the spectrum (2000–1500 cm^−1^) is observed in detail, it can be seen that both the pure polymer and a film made only from the polymer showed peaks at 1699 cm^−1^, which can be assigned to the acid carbonyl groups. When the polymer was mixed with PEG 200 Da, the peak at 1699 shifted to 1713 cm^−1^, indicating crosslinking by an esterification reaction between acid groups in Gantrez^®^ S-97 and the terminal hydroxyl groups from the PEG chains. The same peak is appreciated when both CTAB and PEG GnRs where included in the formulation, suggesting that their presence did not affect the crosslinking process. Peaks at 1773 and 1849 cm^−1^ could be attributed to the formation of anhydride groups between adjacent acid groups in Gantrez^®^ S-97. Furthermore, films were immersed in NaOH 5M, dried, and the IR spectrum was once again recorded (Figure 9B). The same aforementioned peak displacement from 1699 to 1713 cm^−1^ could be seen, but peaks at 1849 and 1773 cm^−1^ disappeared and were replaced by one at 1563 cm^−1^, confirming the formation of anhydride groups when films were crosslinked, which then turned into sodium salts [40,41].

**Figure 10:**
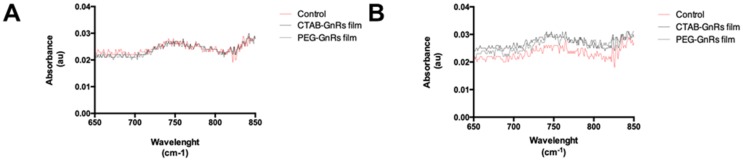
Absorbance of PBS after polymeric films were immersed for 5 min (**A**) and 1 h (**B**).

Approaches where the potential of GnRs can be used without exposing the body directly to them should be followed while strong evidence regarding their safety and toxicity is acquired. By including GnRs into a polymeric crosslinked network, there is a high probability of entrapping the particles within. To test this, films were immersed in PBS and left for 5, 15, 30, and 60 min. At each time, films were removed, and the absorbance spectrum of PBS was directly measured, as shown in Figure 10.

It can be seen that release of GnRs cannot be differentiated from the control film, as in all cases the maximum absorbance is lower than 0.03 au after 5 min of film immersion on PBS. To further explore this, films were kept up to 1 h and PBS absorbance was measured. It can be seen that no significant changes in absorbance were seen, despite the films being partially disintegrated, as seen before with the swelling studies. This could mean that films are indeed disintegrating due to the excess of PBS, however GnRs remain fully entrapped in the crosslinked network and are not released. Even though the study does not replicate what would exactly happen when a film is placed on top of neonate porcine skin, since an excess of water was used, this supports the idea that GnRs could not be markedly released.

### 3.3. Thermal Studies of GnR-Loaded Polymeric Films

**Figure 11:**
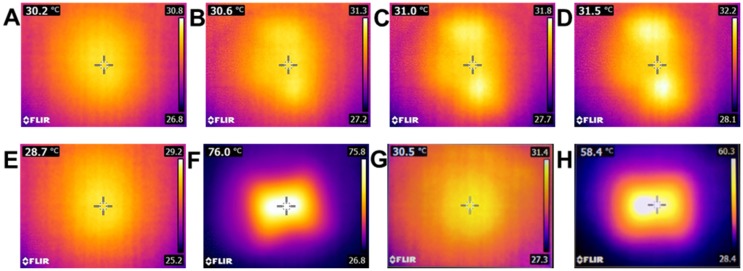
Representative images of temperatures reached by polymeric films with and without GnRs, irradiated with the near-infrared NIR laser. Control film, room temperature and no irradiation (**A**). Control film after 10 s of irradiation (**B**). Control film after 20 s of irradiation (**C**). Control film after 30 s of irradiation (**D**). CTAB-GnRs film, no irradiation (**E**). CTAB-GnRs film irradiated for 15 s (**F**). PEG-GnRs film, no irradiation (**G**). PEG-GnRs film irradiated for 15 s (**H**).

Films were exposed to irradiation using a laser with 2 W power and a wavelength of 808 nm. Studies were carried out using polymeric films with and without GnRs to determine if the polymeric formulation by itself had a certain degree of thermal conductivity upon laser irradiation. Representative thermal images of the films before and after irradiation are shown in Figure 11.

**Figure 12:**
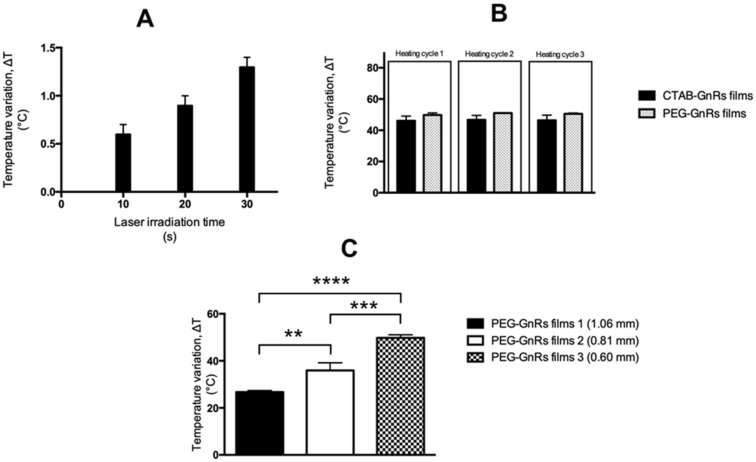
Temperature increase of polymeric film without GnRs irradiated for different times (**A**). Temperature increase of polymeric films prepared with CTAB and PEG-GnRs. Each film was irradiated for cycles of 15 s, cooled down, and repeated two more times (**B**). Temperature increase of polymeric films with different thicknesses, prepared using PEG-GnRs (**C**) (mean ± SD, n = 3) (** *p* = 0.0039, *** *p* = 0.0004, **** *p* < 0.0001).

Films without GnRs exhibited a minimum degree of heating capacity, as even after 30 s of irradiation the temperature increase was below 2 °C (Figure 12A). On the other hand, GnRs-loaded films showed a significant increase in temperature. To achieve local hyperthermia, and therefore a biological response of cell death or regeneration, heating cycles may be needed in order to maintain a temperature over 43 °C for a period of time [42,43]. Therefore, irradiation of polymeric films was conducted for 3 cycles of 10 s each, followed by cooling until room temperature, and thermal images were taken before and after this to calculate the increase in temperature. In all cases, a similar temperature increase was achieved for the GnR-loaded films, fluctuating around 40 °C (Figure 12B). Comparisons between each heating cycle for each type of film (either CTAB-GnRs or PEG-GnRs films) showed no statistical differences; hence, films could be irradiated at least three times without increasing or decreasing the temperature that was initially reached. In addition, comparison between CTAB-GnRs and PEG-GnRs at each heating cycle showed no differences either, suggesting that both films can be heated up and can reach the same temperature. It is surprising that both films reached similar temperatures though, since it was shown in the ICP measurements that the suspension of PEG-GnRs had a lower gold concentration than the CTAB one. Hence, PEG-GnRs films had lower concentrations of GnRs. This could suggest that GnR concentration is not the only factor affecting thermal capacity, but also film density, transparency, and thickness, among others. Indeed, the effect of film thickness was further investigated with films with different thicknesses by pouring decreasing amounts of a gel (and so decreasing amount of GnRs) made with PEG-GnRs onto the silicone release liner. As a result, films with thicknesses of 1.06 ± 0.02, 0.81 ± 0.02, and 0.60 ± 0.08 mm (mean ± SD, n = 3) were obtained and their thermal capacities were compared, as shown in Figure 12C. Despite the fact that the thicker film had a higher concentration of GnRs, there was a clear trend between temperature increase and film thickness: the thinner the films, the higher the temperature reached. This could be explained by the laser mainly irradiating the surfaces of the films, and since the structure was so dense and full of GnRs, the laser could not diffuse into the entire exposed film area, affecting the temperature.

### 3.4. Heating Capacity of Polymeric Films on Neonate Porcine Skin

**Figure 13:**
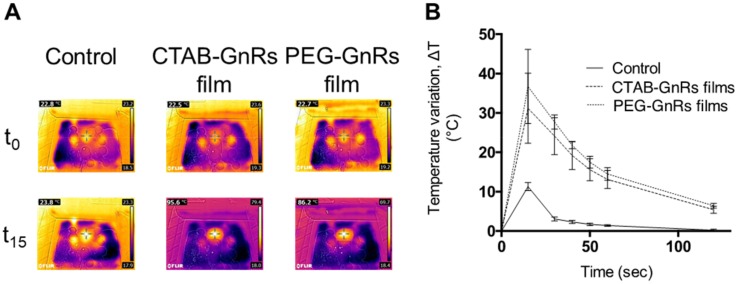
Heating capacity of polymeric films loaded with GnRs on neonate porcine skin. Representative thermal images of films on top of skin after 15 s of laser irradiation (**A**). Temperature increase of porcine skin measured with a thermocouple (mean ± SD, n = 3) (**B**).

To prove the potential of films to heat up a biological tissue, two pieces of neonate porcine skin facing each other’s dermis were used. To control the temperature reached on the bottom of the skin, a thermocouple wire was used and the temperature recorded before and after irradiation. Figure 13 shows representative thermal images, as well as temperature increase data for the films tested. It can be seen in Figure 13A that control film on top of neonate porcine skin heated up by 1 °C after 15 s of irradiation, which correlates to control films being heated only in air, as shown before. On the other hand, porcine skin increased its temperature by around 10 °C (Figure 13B). This could be attributed to the laser heating the porcine skin up and the film working as a “blanket”, keeping the heat. However, after 15 s of switching off the laser, temperature variation dropped to only 3 °C. When either CTAB or the PEG GnR-loaded films were used, a significant increase in temperature was reached after 15 s of irradiation, showing the maximum temperature difference of around 35–45 °C of increase. After switching off the laser, the temperature variation started to drop, but in a very slow manner. After the laser had been switched off, temperature increase ranged between 20 and 30 °C, and 45 s after, the temperature was still over 12 °C. Even after almost 2 min of the laser being switched off, the temperature increase of porcine skin was about 6 °C. It is important to mention that in all cases, even though 1 mL of PBS was placed on top of the skin to keep it moist during the studies and to induce contact between the tissue and the films, it did not break during or after laser irradiation.

## 4. Conclusions

This study demonstrates the potential of GnR-loaded polymeric films to induce superficial hyperthermia upon laser irradiation in a skin model. Inclusion of CTAB and PEG-GnRs in polymeric films did not affect the crosslinking process, or the mechanical or swelling properties. Films could also be cast (or cut before crosslinking) into molds according to the size and shape of skin lesions, offering a tailored treatment for each patient. Body exposure to GnRs will be limited as particles are entrapped in the polymeric network and are not significantly released after swelling of films. This improves the biocompatibility of the material, which is further enhanced by the replacement of CTAB with mPEG-SH. Moreover, as films did not adhere to neonate porcine skin, neither polymeric nor plasmonic material was left behind on the skin.

Heating studies showed that the material’s temperature went up to around 45 °C when films were irradiated with the laser for 15 s, showing the effect of GnRs inclusion on the films, since the temperature of control films did not increase by more than 1.5 °C, even after 30 s of irradiation. Contrary to what it was expected, it is important to mention film thickness played a major role in the heating capacity and temperature increase in comparison to the GnR cargo. When films were placed on top of neonate porcine skin, the increase of temperature of the skin model went up to around 40 °C using either CTAB or PEG-GnRs.

These results prove the use of an innovative approach that combines the plasmonic properties of GnRs and a crosslinked hydrogel for less invasive use of GnRs, avoiding injection of the particles and uncertainty regarding their metabolism, safety, and toxicity once they reach systemic circulation. Future studies should focus on studying the effects of GnR-loaded films on murine skin models of non-melanoma skin cancer, addressing the temperature increase and irradiation time needed to induce an apoptotic response in each case.

## Figures and Tables

**Table 1:** Physical characterization of GnRs (mean ± SD, n = 5).

Sample	Length (nm)	Width (nm)	Aspect Ratio
CATB-GnRs	53.91 ± 7.45	17.70 ± 2.69	3.11 ± 0.73
PEG-GnRs	53.00 ± 7.50	15.02 ± 2.32	3.60 ± 0.78

**Table 2:** Physical characterization of films loaded with GnRs (mean ± SD, n = 3).

Sample	Thickness (mm)	Area (cm^2^)	Weight (g)
CTAB-GnRs	0.61 ± 0.12	1.40 ± 0.18	0.10 ± 0.03
PEG-GnRs	0.60 ± 0.08	1.80 ± 0.17	0.12 ± 0.01
Control	0.64 ± 0.05	1.26 ± 0.08	0.11 ± 0.01
